# The complex becomes more complex: protein-protein interactions of SnRK1 with DUF581 family proteins provide a framework for cell- and stimulus type-specific SnRK1 signaling in plants

**DOI:** 10.3389/fpls.2014.00054

**Published:** 2014-02-21

**Authors:** Madlen Nietzsche, Ingrid Schießl, Frederik Börnke

**Affiliations:** ^1^Division of Biochemistry, Department of Biology, Friedrich-Alexander-Universität Erlangen-NürnbergErlangen, Germany; ^2^Plant Metabolism Group, Leibniz-Institute of Vegetable and Ornamental Crops (IGZ)Großbeeren, Germany; ^3^Institute of Biochemistry and Biology, University of PotsdamPotsdam, Germany

**Keywords:** *Arabidopsis*, SnRK1, DUF581, protein-protein interaction, stress signaling, ABA

## Abstract

In plants, SNF1-related kinase (SnRK1) responds to the availability of carbohydrates as well as to environmental stresses by down-regulating ATP consuming biosynthetic processes, while stimulating energy-generating catabolic reactions through gene expression and post-transcriptional regulation. The functional SnRK1 complex is a heterotrimer where the catalytic α subunit associates with a regulatory β subunit and an activating γ subunit. Several different metabolites as well as the hormone abscisic acid (ABA) have been shown to modulate SnRK1 activity in a cell- and stimulus-type specific manner. It has been proposed that tissue- or stimulus-specific expression of adapter proteins mediating SnRK1 regulation can at least partly explain the differences observed in SnRK1 signaling. By using yeast two-hybrid and *in planta* bi-molecular fluorescence complementation assays we were able to demonstrate that proteins containing the domain of unknown function (DUF) 581 could interact with both isoforms of the SnRK1α subunit (AKIN10/11) of *Arabidopsis*. A structure/function analysis suggests that the DUF581 is a generic SnRK1 interaction module and co-expression with DUF581 proteins in plant cells leads to reallocation of the kinase to specific regions within the nucleus. Yeast two-hybrid analyses suggest that SnRK1 and DUF581 proteins share common interaction partners inside the nucleus. The analysis of available microarray data implies that expression of the 19 members of the *DUF581* encoding gene family in *Arabidopsis* is differentially regulated by hormones and environmental cues, indicating specialized functions of individual family members. We hypothesize that DUF581 proteins could act as mediators conferring tissue- and stimulus-type specific differences in SnRK1 regulation.

## Introduction

The maintenance of cellular energy homeostasis in response to fluctuating internal and external conditions is vital for all living organisms. In eukaryotes, an evolutionarily conserved protein kinase known as AMP-activated protein kinase (AMPK) in animals, sucrose non-fermenting kinase 1 (SNF1) in yeast, and SNF1-related protein kinase 1 (SnRK1) in plants integrates environmental stress signals, nutrient availability and energy depletion into adaptational responses (Hardie, 2007; Halford and Hey, 2009; Ghillebert et al., 2011). These include down-regulation of ATP-consuming processes and induction of energy-generating catabolic reactions through post-translational modification of key metabolic enzymes as well as large-scale transcriptional reprogramming (Baena-González et al., 2007; Baena-González and Sheen, 2008). In addition, AMPK, SNF1, and SnRK1 are also required for the regulation of storage carbohydrate accumulation in their respective system (Halford and Hey, 2009). The catalytic AMPK/SNF1/SnRK1 α subunit is typically organized into a heterotrimeric complex consisting of additional regulatory β and γ subunits (Hardie, 2007; Polge and Thomas, 2007; Ghillebert et al., 2011). Similar to its counterparts in animals and yeast, SnRK1 activity is regulated by phosphorylation/de-phosphorylation of a T-loop threonine (Thr172 of *Arabidopsis thaliana* SnRK1.1α) involving upstream kinases SnaK1/2 for activation (Shen and Hanley-Bowdoin, 2006; Hey et al., 2007; Shen et al., 2009; Crozet et al., 2010) and the PP2C phosphatases ABI1 and PP2CA for inactivation (Rodrigues et al., 2013). In addition, SnRK1 activity is prone to allosteric regulation by several metabolites. Glucose-6-phosphate has been shown to inhibit SnRK1 activity in all tissues tested when applied in millimolar concentrations (Toroser et al., 2000; Zhang et al., 2009; Nunes et al., 2013). Recently, it was shown that trehalose-6-phosphate (T6P), the direct precursor of trehalose in plants, inhibits SnRK1 in physiological amounts (1–100 μM) as well as in a tissue and developmental stage specific manner (Zhang et al., 2009; Debast et al., 2011; Martínez-Barajas et al., 2011). T6P levels in *Arabidopsis* seedlings have been shown to correlate with sucrose content, leading to the proposal that T6P acts as a signal of sucrose status in plants (Lunn et al., 2006; O'Hara et al., 2013). Thus, inhibition of SnRK1 by T6P couples carbohydrate availability for metabolism and growth with starvation signaling (O'Hara et al., 2013). Experimental evidence suggests that SnRK1 inhibition by T6P is dependent on an additional protein factor that can be separated from the catalytic α subunit and which is only present in growing tissue such as seedlings, young leaves, growing potato tubers, and developing wheat grain (Zhang et al., 2009; Debast et al., 2011; Martínez-Barajas et al., 2011). Apart from being regulated by sugar signals, SnRK1 also responds to hormonal signals, in particular to abscisic acid (ABA), possibly linking hormone and sugar signaling pathways (Radchuk et al., 2006, 2010; Jossier et al., 2009; Coello et al., 2012; Tsai and Gazzarrini, 2012; Rodrigues et al., 2013). In addition to metabolic readjustment, SnRK1 coordinates the responses to a wide array of stresses such as flooding, sudden darkness, salinity, and pathogen attack (Hao et al., 2003; Lovas et al., 2003; Schwachtje et al., 2006; Baena-González et al., 2007; Lee et al., 2009). Although SnRK1 obviously acts as a convergent point for many different environmental and metabolic signals to control growth and development, it is currently unknown how these many different signals could be translated into a cell-type or stimulus specific response.

Plants generally encode several isoforms of each SnRK1 subunit with environmentally controlled and developmental stage or tissue-specific expression patterns (Polge and Thomas, 2007; Polge et al., 2008). Thus, various non-catalytic subunits may be available depending on the conditions or the cell type, allowing SnRK1 to respond to various stimuli. For example, the kinase could respond to ABA by a modification in the composition of the complex, as the LeSNF4 γ subunit in tomato is induced by ABA treatment (Bradford et al., 2003).

The two α subunit isoforms [SnRK1.1/AKIN10 (At3g01090) and SnRK1.2/AKIN11 (At3g29160)] from *Arabidopsis* have been shown to orchestrate transcriptional reprogramming of approximately 1000 genes in the acclimation response to energy deficit (Baena-González et al., 2007). Thus, the kinase likely regulates gene expression in the nucleus by phosphorylation-mediated activity modulation of transcription factors. The broad effect on transcription and the specificity that is required to respond to a particular stress is likely to require modulation of a range of different downstream target proteins. However, only a few have yet been identified (Baena-González et al., 2007; Hanson et al., 2008; Coello et al., 2012; Tsai and Gazzarrini, 2012; Chen et al., 2013b). It is currently unknown whether heterogeneity of SnRK1 complex subunit composition can modulate substrate specificity of the kinase or whether additional protein factors are recruited to the SnRK1 complex under certain conditions which then could mediate interaction with specific target proteins to enable context specific regulation.

In the present study, we identified domain of unknown function (DUF) 581 containing proteins as interaction partners of AKIN10 and AKIN11. A structure/function analysis suggests that the DUF581 is a generic SnRK1 interaction module. Co-expression of SnRK1 with DUF581 proteins leads to reallocation of the kinase to specific regions within the nucleus. Yeast two-hybrid analyses indicate that SnRK1 and DUF581 proteins can share common interaction partners inside the nucleus. A model in which DUF581 proteins mediate the interaction of SnRK1 with specific target proteins is discussed.

## Materials and methods

### Plasmid construction

The entire coding region of DUF581 protein encoding genes as well as of SnRK1.1/AKIN10 and SnRK1.2/AKIN11 was amplified by polymerase chain reaction from *Arabidopsis* cDNA using the primers listed in Supplementary Table S2. The resulting fragments were inserted into the pENTR-D/TOPO vector according to the manufacturer's instructions (Invitrogen) and verified by sequencing. For yeast two-hybrid analysis, fragments were recombined into Gateway®-compatible versions of the GAL4-DNA binding domain vector pGBT-9 and the activation domain vector pGAD424 (Clontech) using L/R-clonase (Invitrogen). To generate translational fusions between DUF581 proteins and the green fluorescent protein (GFP) coding sequences were inserted into the vector pK7FWG2 (Karimi et al., 2002). AKIN10/11-mCherry fusions are based on the pRB-GW-mCherry vector and were generated by Gateway®-cloning. Constructs for bi-molecular complementation analysis are based on Gateway®-cloning compatible versions of pRB-C-Venus^N173^ and pRB-C-Venus^C155^.

### Site directed mutagenesis

Site directed mutagenesis of DUF581 constructs was carried out using the Quick-change site directed mutagenesis kit (Stratagene, Heidelberg, Germany) employing primers listed in Supplementary Table S2 online. All base changes were verified by sequencing.

### Yeast two-hybrid analyses

Yeast two-hybrid techniques were performed according to the yeast protocols handbook and the Matchmaker GAL4 Two-hybrid System 3 manual (both Clontech, Heidelberg, Germany). Direct interaction of two proteins was investigated by cotransformation of the respective plasmids in the yeast strain Y190, followed by selection of transformants on medium lacking Leu and Trp at 30°C for 3 days and subsequent transfer to medium lacking Leu, Trp and His (supplemented with 25 mM 3-amino-triazole) for growth selection and *lacZ* activity testing of interacting clones.

### Agrobacteria-infiltration

For infiltration of *Nicotiana benthamiana* leaves, *A. tumefaciens* C58C1 carrying the construct of interest was infiltrated into the abaxial air space of 4- to 6-week-old plants, using a needleless 2-ml syringe. Agrobacteria were cultivated overnight at 28°C in the presence of appropriate antibiotics. The cultures were harvested by centrifugation, and the pellet was resuspended in sterile water to a final optical density at (OD_600_) of 1.0. The cells were used for the infiltration directly after resuspension.

### Confocal microscopy and BiFC assay

Localization experiments and BiFC were performed in Agro-infiltrated leaves of *N. benthamiana* 48 h post infiltration as described previously using a Leica TCS SP5II (Arsova et al., 2010).

## Results

### The DUF581 protein family in *arabidopsis*

**Figure 1:**
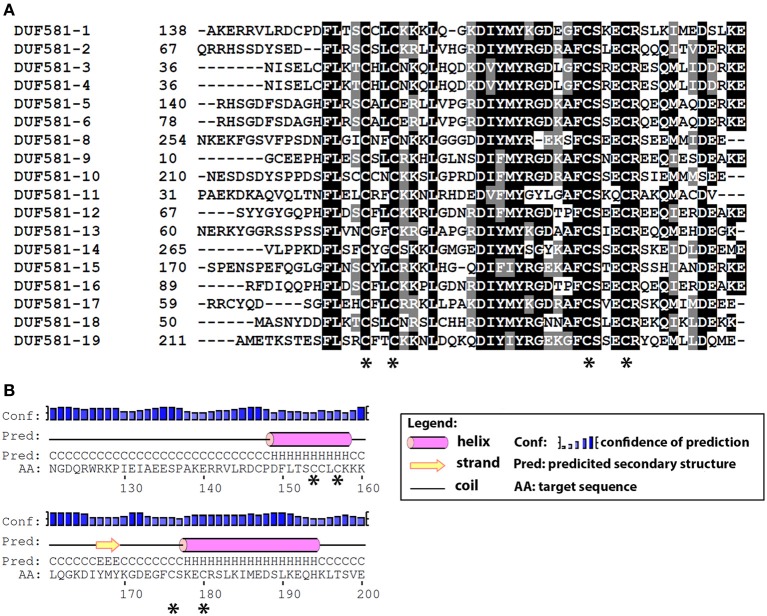
**Sequence alignment and predicted topology of the DUF581. (A)** Multiple alignment of the DUF581 from DUF581 containing proteins of *Arabidopsis*. Sequences were aligned using CLUSTALW and displayed by using BOXSHADE (www.ch.embnet.org/software/BOX_form.html). The conserved cysteine residues are indicated by an asterisk. **(B)** Topology prediction of the DUF581 using the Jpred tool (Cole et al., 2008). The region comprising the DUF581 of DUF581-1 was chosen as a representative. The conserved cysteines are indicated by an asterisk.

A high-throughput yeast-two-hybrid screen, that defined a proteome-wide binary protein-protein interaction map for the interactome network of the plant *Arabidopsis thaliana*, identified a number of DUF581 containing proteins as potential interaction partners of the SnRK1 isoforms SnRK1.1/AKIN10 (At3g01090) and SnRK1.2/AKIN11 (At3g29160), respectively (*Arabidopsis* Arabidopsis Interactome Mapping Consortium, 2011). In *Arabidopsis*, 19 genes encode predicted proteins that contain a DUF581 (Supplementary Table S1), identified here as DUF581-1 to DUF581-19. These proteins range from 92 to 344 amino acids in size while the DUF581 itself comprises 49–54 amino acids (Supplementary Figure S1). Two double cysteine motifs interspersed by 19 amino acids (C-X2C-X8-D-X3-Y-X5-F-CSXE/QCR) are highly conserved within the DUF581 domain (Figure 1A). A secondary structure prediction suggests that the two double cysteine motifs are organized in α-helices interspersed by a short β-strand (Figure 1B). One protein annotated as a DUF581 family member, DUF581-7 appears to lack the characteristic features of the domain and was thus not included into the multiple sequence alignment as it is likely not a member of this protein family. DUF581-3 and -4 have identical protein sequences and it is currently unclear whether the two annotated loci represent two genes. With the exception of DUF581-11, expression of all family members is supported by cDNAs (Supplementary Table S1). Thus, DUF581-11 is either a pseudogene or expressed in a very narrow range of cell types.

Outside the DUF581, family members shared only little similarity on the polypeptide level (Supplementary Figure S2). A search of the Pfam protein family database (www.pfam.sanger.ac.uk) revealed that DUF581 proteins are confined to the plant kingdom, indicating a plant specific function of these proteins. The number of family members varies between species ranging from only one clearly identifiable DUF581 protein in the moss *Physcomitrella patens* to 16 members in *Vitis vinifera* and *Ricinus communis*. The monocots Rice *(Oryza sativa*) and Maize (*Zea mays*) possess 33 and 50 members, respectively, a particularly high number of DUF581 containing proteins. This suggests an evolutionary amplification of DUF581 proteins and an expansion into a multi-member protein family in higher plants.

**Figure 2:**
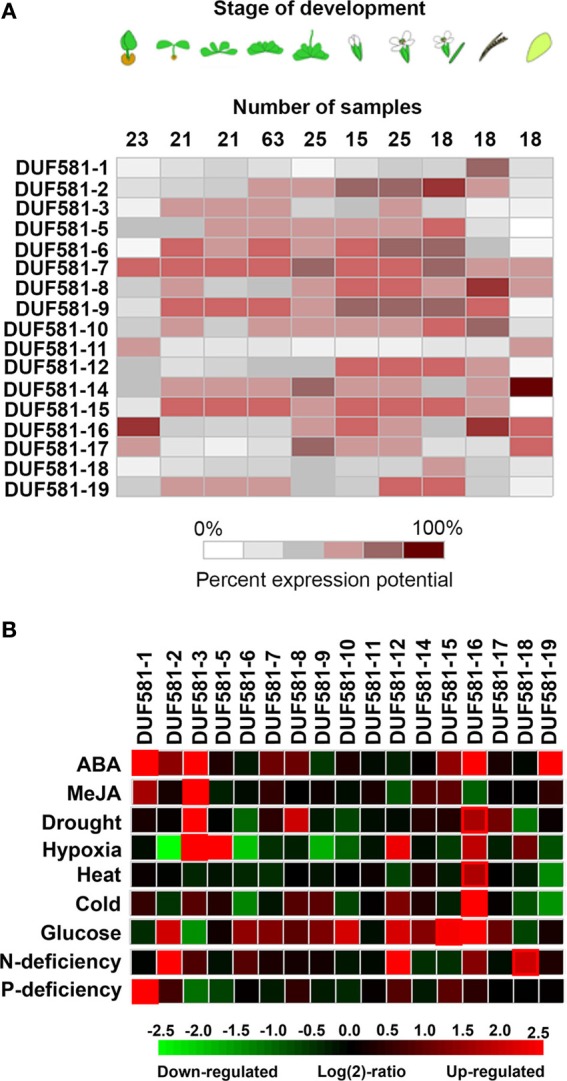
**Heat map comparing expression of *Arabidopsis* DUF581 protein encoding genes using microarray data available in the Genevestigator database (Hruz et al., 2008). (A)** Developmental time course of *DUF581* gene expression in *Arabidopsis*. Datasets derived from the analysis of tissue-specific gene expression or developmental time courses of Col-0 plants were used for the meta-analysis. **(B)** Impact on different perturbations on *DUF581* expression level. ABA; excised leaf samples of 5 weeks old *Arabidopsis thaliana* Col-0 wild-type plants were treated with 50 mM ABA for 3 h (experiment ID AT-000433). MeJa; Cell samples of T87 cell suspension that was grown under 16 h light/8 h dark cycles were exposed to 100 μ M MeJa (methyl jasmonate) for 24 h (experiment ID-AT000539). Drought; green tissues of treated seedlings were harvested 24 h after onset of treatment (experiment ID AT-000419). Heat; seedling samples of Col grown for 11 days on peat pellets at 21°C were transferred to 40°C for 1h (experiment ID AT-000645). Cold; 16-day old seedlings were transferred to 4°C under low light and green tissues were harvested after 24 h (experiment ID AT-00120). Glucose; seedling samples of Col grown for 5 days on solid medium (0.5x Murashige and Skoog (MS) salts, 1% sucrose, 0.8% agar) were washed seven times with water and once with liquid medium (0.5x MS salts), incubated for 24 h in liquid medium and then incubated in liquid medium containing 3% glucose and for 3 h in darkness (experiment ID AT-000650). N-deficiency; seedling samples of Col-0 grown in sterile liquid medium with 4 mM KNO_3_ (N-replete condition) for 7 days and then for 2 days in medium with no N added (N-deplete condition) (experiment ID AT-000405).

In order to analyze the expression pattern of individual DUF581 protein encoding genes in *Arabidopsis*, we carried out a meta-analysis of selected publicly available microarray data sets lodged with the Genevestigator database (www.genevestigator.com; Hruz et al., 2008). Expression of DUF581 genes varied across developmental stages (Figure 2A) with most family members being expressed during the vegetative stage as well as during the reproductive stage. Some *DUF581* genes (e.g., *DUF581-2, -6, -9*, and *-10*) appear to be more abundantly expressed in flower organs than in green tissues. DUF581-1, -11, and -18 show highest expression in green/mature siliques, seeds and seedlings with almost no expression in leaf tissues. Especially *DUF581-11* appears to be expressed in seed only, providing an explanation for the absence of cDNAs. This might indicate a specialized function of these family members in the respective tissues.

Expression of the *DUF581* genes in *Arabidopsis* is highly responsive to hormones and environmental cues (Figure 2B). Several members are strongly induced by the external application of ABA (*DUF581-1, -2, -3, -16*, and *-18*) or methyl-jasmonate (MeJA; *DUF581-3*). In addition, abiotic stress had strong influence on *DUF581* expression. *DUF581-3* and *-8* are induced under drought conditions. Interestingly, under hypoxic conditions individual *DUF581* genes display contrasting expression patterns, with *-3* and *-4* being strongly induced, while *-2*, *-6*, and *-9* are considerably repressed (Figure 2B). *DUF581-16* showed higher expression levels under heat as well as under cold treatment. The availability of nutrients also has great impact on *DUF581* expression. External application of glucose leads to induction of, e.g., *DUF581-15* and *-16* while *DUF581-3* is down-regulated under these conditions. Nitrogen deprivation induces expression of *DUF581-2*, *-12*, and *-18* and phosphate starvation specifically leads to the induction of *DUF581-1*.

Taken together, *DUF581* expression is highly responsive to a number of perturbations aligning with conditions under which SnRK1 signaling is supposed to play an important role (Coello et al., 2011).

### Domain of unknown function (DUF) 581 containing proteins interact with SnRK1 in a yeast two-hybrid assay

**Figure 3:**
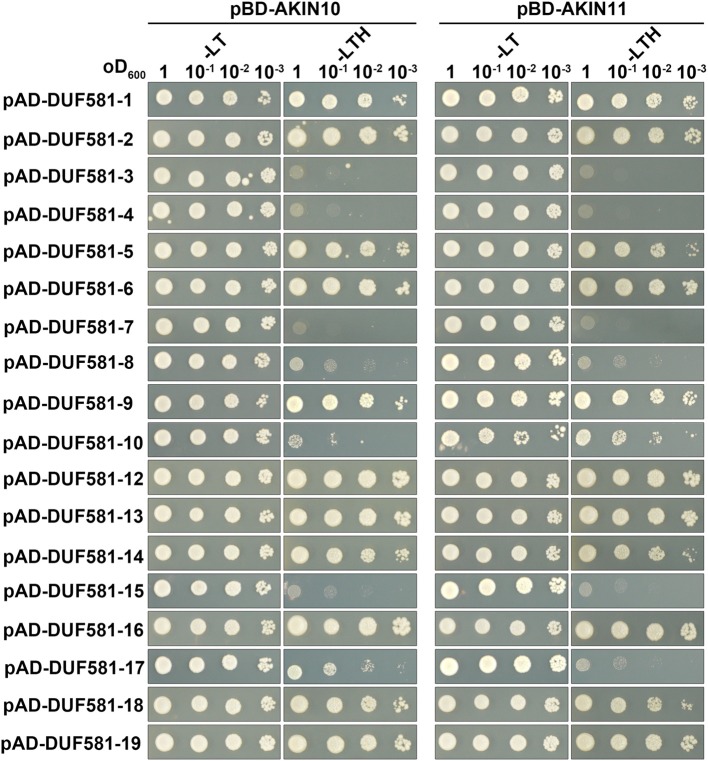
**Interaction of AKIN10 and 11 with *Arabidopsis* DUF581 proteins in yeast.** *Arabidopsis* DUF581 proteins fused to the GAL4 DNA-binding domain (BD) were expressed in combination with either AKIN10 or AKIN11 fused to the GAL4 activation domain (AD) in yeast strain Y190. Cells were grown on selective media for 3 days at 30°C before the pictures were taken. –LT, yeast growth on medium without Leu and Trp. –LTH, yeast growth on medium lacking Trp, Leu, and His, indicating expression of the *HIS3* reporter gene.

Given the frequency DUF581 proteins appeared as AKIN10/AKIN11 interaction partners in a high-throughput yeast-two-hybrid screen, we hypothesized that this domain could function as a SnRK1 interaction domain on a more general basis. In order to experimentally test this hypothesis in a systematic fashion, all DUF581 family members, except DUF581-11, were cloned from *Arabidopsis* and tested for the ability to interact with AKIN10 and AKIN11 in yeast. To this end, DUF581 proteins fused to the GAL4 activation domain were transformed in pairwise combinations with either AKIN10 or AKIN11 fused to the GAL4 binding domain into a yeast reporter strain. Interaction between two given proteins was visualized by monitoring growth on medium lacking histidine. As shown in Figure 3, the majority of DUF581 proteins tested were able to interact with both *Arabidopsis* SnRK1 isoforms, although to a different extent. Only DUF581-3, -4, -7, and -15 did not restore growth in the absence of histidine and thus do not seem to interact with SnRK1 in yeast. In order to investigate whether DUF581 proteins from another species would also bind to SnRK1 in yeast, we cloned a DUF581 protein coding fragment from potato which shares approximately 56% overall similarity with *Arabidopsis* DUF581-6. A direct interaction test of the potato DUF581 protein with potato SnRK1 revealed that both proteins interact in yeast (Supplementary Figure S3). Thus, we conclude that DUF581 containing proteins from different plants are able to interact with SnRK1 in yeast.

**Figure 4:**
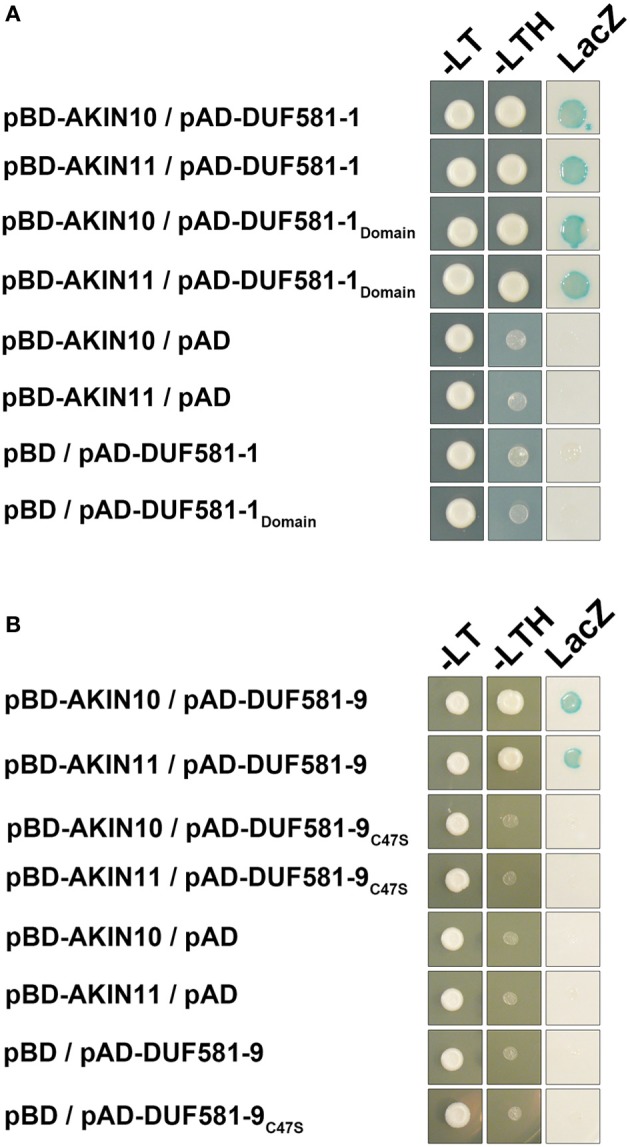
**DUF581 is necessary and sufficient to mediate SnRK1 binding in yeast.** Cells were grown on selective media for 3 days at 30°C before the pictures were taken. Empty BD and AD vectors served as control. –LT, yeast growth on medium without Leu and Trp. –HLT, yeast growth on medium lacking His, Leu, and Trp, indicating expression of the *HIS3* reporter gene. LacZ, activity of the *lacZ* reporter gene. **(A)** AKIN10 or AKIN11 fused to the GAL4 DNA-binding domain (BD) were expressed in combination with truncation construct of DUF581-1 comprising the DUF581 (amino acids 138–192) fused to the GAL4 activation domain (AD) in yeast strain Y190. **(B)** AKIN10 or AKIN11 fused to the GAL4 DNA-binding domain (BD) were expressed in combination with pAD-DUF581-9C47S, carrying a cysteine to serine exchange within the DUF581.

Because *Arabidopsis* DUF581 proteins share relatively little similarity on the polypeptide level outside the DUF581 itself, we anticipated that interaction with SnRK1 is mediated by this particular protein domain. That said, a deletion construct of DUF581-1 comprising amino acids 138–192 representing the entire DUF581 was generated as a GAL4 activation domain fusion and tested for its ability to interact with AKIN10/AKIN11 in yeast. As shown in Figure 4A, the DUF581-1 deletion construct retained its ability to bind AKIN10 in yeast, suggesting that the interaction is mediated by this protein domain. A unifying feature of the DUF581 is the double cysteine motif. In order to investigate whether the integrity of the cysteine motif within the DUF581 is required for SnRK1 binding, the cysteine residue at position 47 of DUF581-9 was changed to a serine by site-directed mutagenesis. When fused to the GAL4 activation-domain, the DUF581-9C47S protein variant lost its ability to interact with SnRK1 in yeast (Figure 4B), strongly suggesting that a functional DUF581 is required for binding.

In summary, the yeast two-hybrid analyses strongly suggest that interaction with SnRK1 is a general feature of DUF581 containing proteins and that the DUF581 is required and sufficient to mediate this interaction.

### *arabidopsis* DUF581 proteins localize to cytosol and the nucleus when transiently expressed in *N. benthamiana*

**Figure 5:**
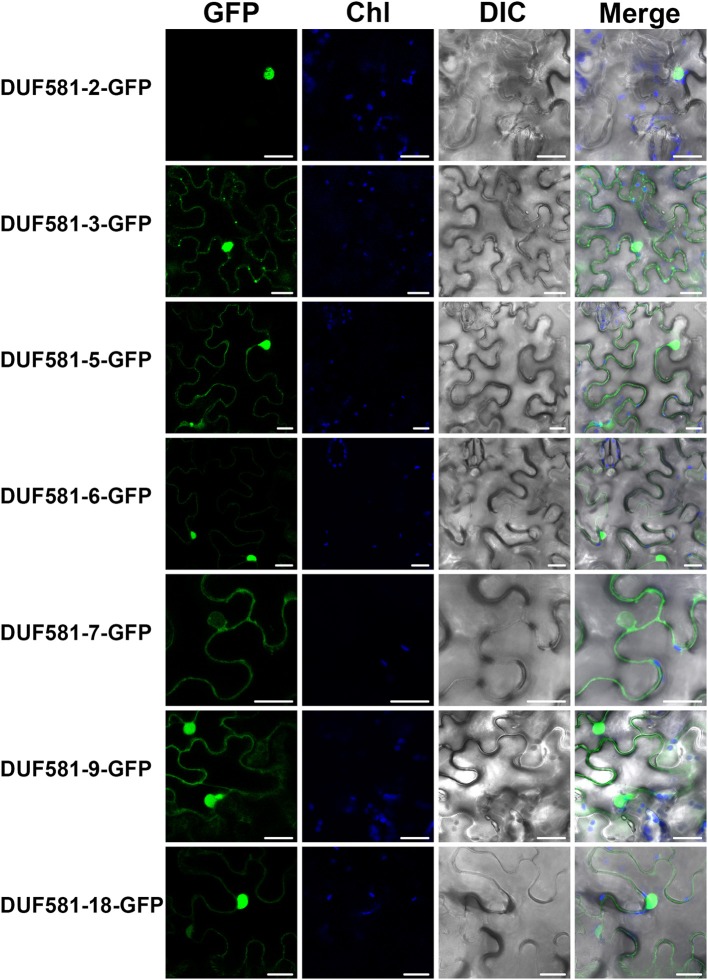
**Subcellular localization of DUF581-GFP proteins.** Subcellular localization of DUF581-GFP tagged proteins in *N. benthamiana* leaves transiently transformed by Agroinfiltration. The green fluorescence (GFP), chlorophyll autofluorescence (Chl) and a brightfield image were recorded and the resulting images were merged. Bars = 20 μm.

To investigate the subcellular localization of *Arabidopsis* DUF581 proteins, we generated GFP C-terminal protein fusions for some DUFs (DUF581-2, -3, -5, -6, -7, -9, -18) under the control of the constitutive Cauliflower mosaic virus 35S (CaMV-35S) promoter and transiently expressed GFP-fusion proteins in leaves of *N. benthamiana* plants using *Agrobacterium tumefaciens* infiltration. Confocal laser scanning microscopy of infiltrated leaves revealed that in almost all cases investigated GFP fluorescence was confined to the nucleus of the cell and to a lesser extent to the cortical cytoplasm of leaf epidermal cells (Figure 5). DUF581-7-GFP formed an exception from the observed pattern in that GFP fluorescence was not detected inside the nucleus, suggesting a cytoplasmic localization of the protein. DUF581-3-GFP additionally showed fluorescence in discrete spots dispersed throughout the cytoplasm (Figure 5). In essence, subcellular localization data of *Arabidopsis* DUF581 proteins using a GFP reporter argue for a nucleo-cytoplasmic distribution of these proteins in plant cells.

### *arabidopsis* DUF581-GFP proteins co-localize with SNRK1-mCherry inside the nucleus

**Figure 6:**
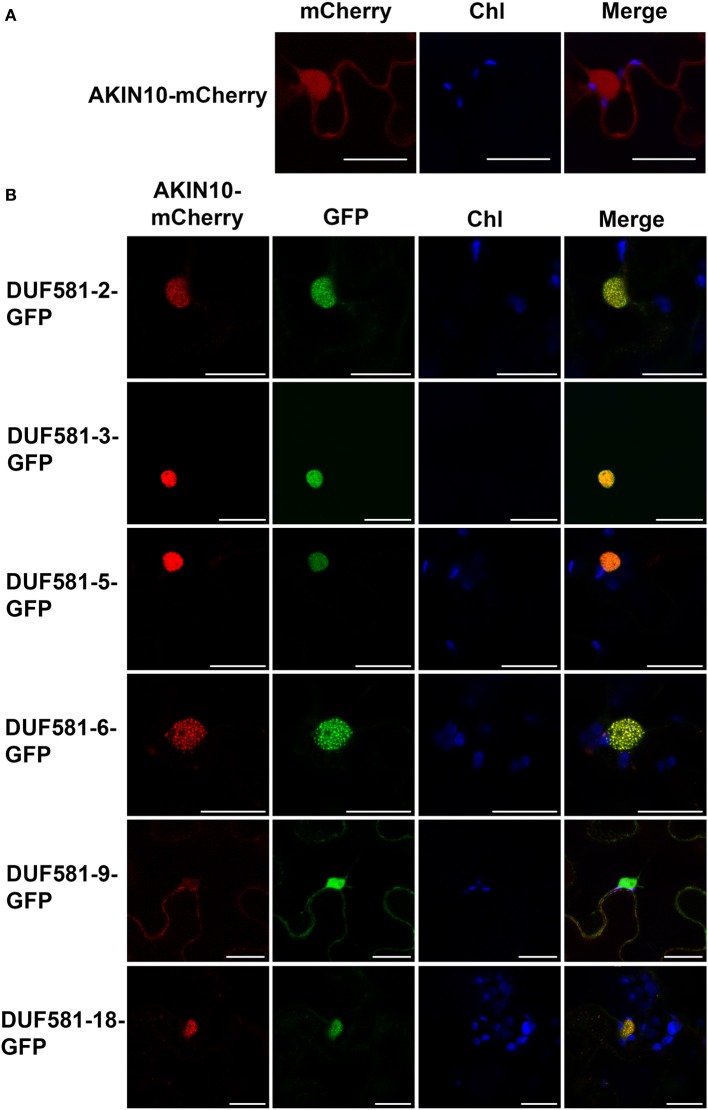
**AKIN10-mCherry and DUF581-GFP fusions co-localize in the nucleus.** Subcellular localization of DUF581-GFP and AKIN10-mCherry fusions in *N. benthamiana* leaves transiently co-transformed by Agro-infiltration. The green fluorescence (GFP), red fluorescence (mCherry) and chlorophyll autofluorescence (Chl) were monitored separately to prevent cross-talk of the fluorescence channels and the resulting fluorescence images were merged. Bars = 20 μm. **(A)** Micrographs of *N. benthamiana* leaf cells expressing AKIN10-mCherry alone. **(B)** Microscopic pictures taken of *N. benthamiana* leaf cells coexpressing AKIN10-mCherry with different members of the DUF581 protein family.

To enable a functional interaction *in planta*, both SnRK1 and DUF581 proteins require an at least partially overlapping subcellular localization. To visualize localization of both types of proteins within the same cell, AKIN10 was fused with mCherry and transiently expressed in leaves of *N. benthamiana* either individually or in combination with a particular DUF581-GFP construct. In consistence with previous findings (Bitrian et al., 2011; Cho et al., 2012) mCherry fluorescence could readily be detected within the cytosol and the nucleus of leaf epidermal cells expressing AKIN10-mCherry alone (Figure 6A). When co-expressed with DUF581 proteins, GFP and mCherry fluorescence co-localized within the nucleus (Figure 6B). Interestingly, with exception of the AKIN10/DUF581-9 combination, the AKIN-mCherry fluorescence appeared to be exclusively localized within the nucleus when a DUF581-GFP protein co-expressed within the same cell. The pattern of AKIN-mCherry fluorescence in DUF581-GFP co-expressing cells implied that SnRK1 is actively shuttled into the nucleus when a particular DUF581 protein is present.

It appeared that in some cases fluorescence of both reporter constructs localized to sub-nuclear foci, which were evenly distributed throughout the nucleus (Figure 6B). This pattern was not observed when either AKIN-mCherry or a DUF581-GFP protein was expressed on its own and thus might be mediated through the interaction between both proteins.

### DUF581 proteins interact with AKIN10/11 within sub-nuclear foci

We further examined the interaction between AKIN10/11 and DUF581-6, -9, or -18, as representatives for this protein family, *in planta* using a bimolecular fluorescence complementation (BiFC) assay via transient expression in leaves of *N. benthamiana* (Walter et al., 2004). In this experiment, two split fragments of the Venus fluorescent protein (N-terminal 173 residues; Venus^N^, and the C-terminal 83 residues; Venus^C^), a variant of enhanced yellow fluorescent protein, were translationally fused to the C-terminus of AKIN10/11 and the C-terminus of DUF581-6/-18, respectively. Fusion constructs were expressed under control of the CaMV35S promoter and interaction between AKIN-Venus^N^ and DUF581-Venus^C^ brings the two non-functional halves of Venus into close proximity to reconstitute a functional fluorophore.

**Figure 7:**
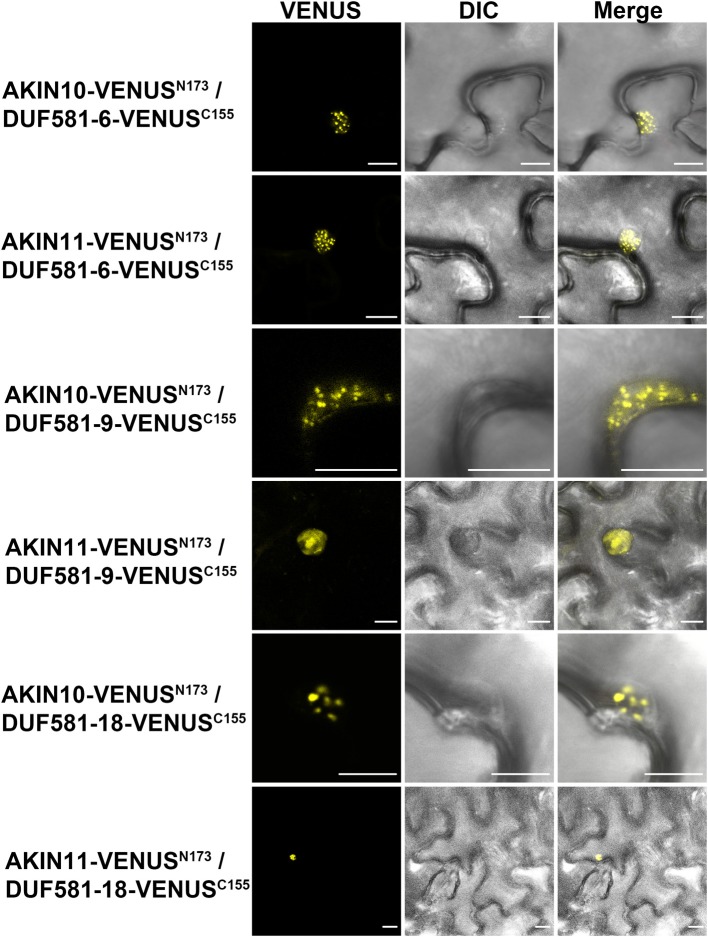
**AKIN10/11 interact with DUF581 proteins in sub-nuclear foci.** Visualization of protein-protein interactions *in planta* by the BiFC assay. Venus confocal microscopy images show *N. benthamiana* leaf epidermal cells transiently expressing constructs encoding the fusion proteins indicated. Bars = 10 μm.

Homodimerization of cytosolic fructose-1,6-bisphosphatase (FBPase) in the cytosol served as a positive control (Supplementary Figure S4). A combination of FBPase-Venus^C^ with AKIN10/11-Venus^N^ induced no fluorescence (Supplementary Figure S4). By contrast, strong Venus fluorescence was observed when combinations of AKIN10/11 and DUF581-6 or DUF581-18 were expressed demonstrating SnRK1/DUF581 complex formation in plant cells (Figure 7). Similar to the fluorescence pattern observed in the co-localization experiments, the fluorescence signal in AKIN/DUF581 BiFC experiments was also detected in specific regions inside the nucleus (Figure 7). Taken together, these results strongly support the hypothesis that SnRK1 and DUF581 proteins interact inside the nucleus within sub-nuclear foci.

### DUF581-18 and AKIN10/11 have a common interaction partner

The data obtained so far are in support of a model in which SnRK1 regulates specific transcriptional changes inside the nucleus during the physiological adaptation of the plant to various stresses (Baena-González et al., 2007; Cho et al., 2012). In this scenario, DUF581 proteins could either act as SnRK1 substrate directly acting as transcriptional regulators or they could act as scaffold proteins bridging the interaction of SnRK1 with a particular substrate protein. None of the DUF581 proteins contains an obvious DNA binding domain or other structural features characteristic for transcription factors; however, most of them possess a highly variable region that could well mediate interaction with a third protein inside the nucleus and thus guide potential substrates for phosphorylation by SnRK1.

**Figure 8:**
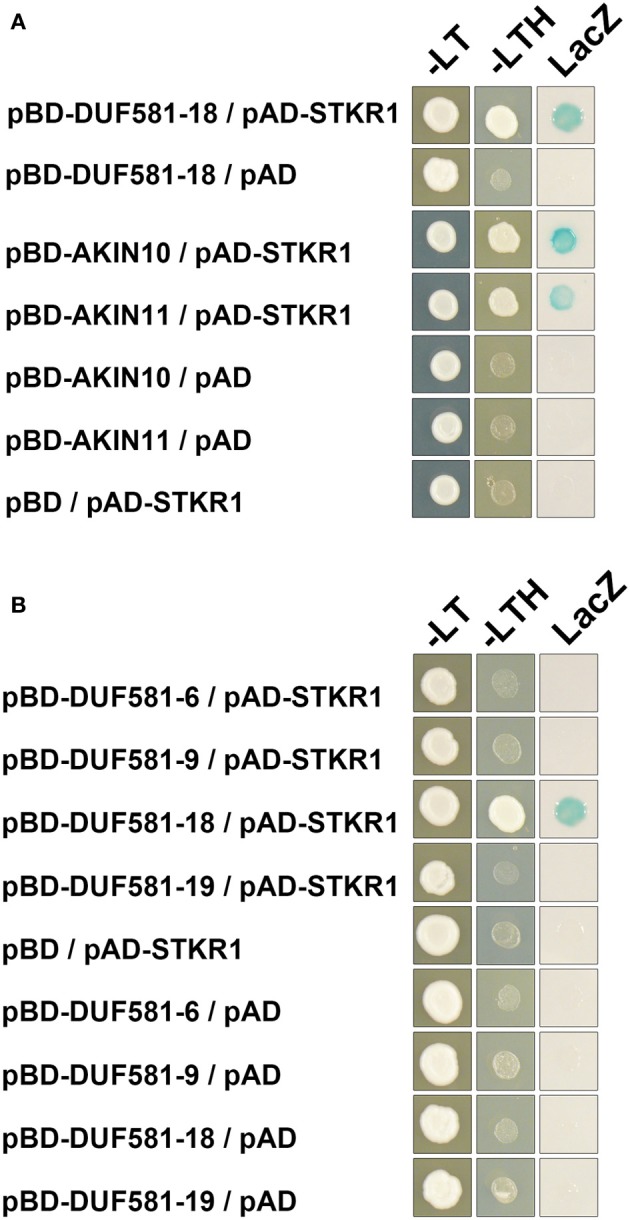
**AKIN10/11 and DUF581-18 share a common interaction partner.** Yeast cells were grown on selective media for 3 days at 30°C before the pictures were taken. Empty BD and AD vectors served as control. –LT, yeast growth on medium without Leu and Trp. –HLT, yeast growth on medium lacking His, Leu, and Trp, indicating expression of the *HIS3* reporter gene. LacZ, activity of the *lacZ* reporter gene. **(A)** AKIN10/11 or DUF581-18 fused to the GAL4 DNA-binding domain (BD) was expressed in combination with STOREKEEPER1 (STKR1) fused to the GAL4 activation domain (AD) in yeast strain Y190. **(B)** Different DUF581 proteins from *Arabidopsis* fused to the GAL4 DNA-binding domain (BD) were expressed in combination with STOREKEEPER1 (STKR1) fused to the GAL4 activation domain (AD).

To further test the hypothesis that DUF581 proteins interact with other nuclear proteins and possibly have shared interacting partners with SnRK1, we performed a blind Y2H library screen using DUF581-18 as a representative member of this protein family. As a proof of concept we were able to identify AKIN10 as an interaction partner of DUF581-18 in the library screen (data not shown). In addition, multiple clones encoded a protein annotated as DNA-binding storekeeper protein-related transcriptional regulator (At4g00270; hereafter referred to as STKR1 for storekeeper related 1), member of a protein family that has been associated with sucrose-regulated gene expression in potato (Zourelidou et al., 2002). A direct interaction test revealed that STKR1 was not only able to interact with DUF581-18 in yeast but also binds to AKIN10 and AKIN11 (Figure 8A). This opens the possibility that all three proteins could assemble into the same complex. Importantly, STKR1 was not able to interact with other members of the DUF581 family, indicating that the interaction with DUF581-18 is rather specific and not mediated by the DUF581 itself (Figure 8B).

## Discussion

SnRK1 protein kinases are considered central regulators of energy and stress signaling in plants. Depending on the tissue or the developmental state, SnRK1 signaling responds to specific stimuli by triggering transcriptional changes inside the nucleus which are generally accompanied by a down-regulation of energy consuming processes and an induction of alternative ATP-producing pathways (Baena-González et al., 2007; Baena-González and Sheen, 2008; Zhang et al., 2009). However, the molecular basis of tissue and stimulus-type specific differences in SnRK1 signaling is currently not well understood. The subunit composition of the SnRK1 complex has been suggested to be one determinant for signaling specificity (Ramon et al., 2013). Besides the canonical KINα, KINβ, and KINγ or KINβγ subunits the complex could contain additional auxiliary proteins that aid recognition of specific phosphorylation substrates under particular internal or external conditions.

We could show that majority of *Arabidopsis* proteins containing DUF581 can interact with SnRK1.1/AKIN10 and SnRK1.2/AKIN11 in yeast and *in planta*. We cautiously speculate that the few examples where we did not find interaction between a particular DUF581 protein and SnRK1 are due to technical limitations of the yeast two-hybrid system or non-functional expression of the protein in yeast. Future experiments will have to address this issue using alternative approaches.

The DUF581 is necessary and sufficient to mediate this interaction and can thus be considered as a SnRK1 adaptor domain (SAD). Loss of SnRK1 interaction of the C47S variant of DUF581-9 suggests that the conserved cysteine residues are important to form a functional SAD. It has previously been suggested that the four cysteines could bind zinc in a tetrahedral coordination (He and Gan, 2004). Thus, DUF581/SAD could represent a novel type of C4-zinc finger domain. C4-zinc fingers are present in a range of eukaryotic as well as prokaryotic proteins although their sequence as well as the distance between the cysteine motifs is not conserved. SAD containing proteins are confined to the plant kingdom and the protein family is considerably expanded in higher plants. Under the assumption that SAD/DUF581 proteins are involved in SnRK1 regulation, the large number of family members in higher plants would reflect the broad range of physiological contexts involving SnRK1 signaling. Expression of SAD/DUF581 protein encoding genes is highly responsive to hormones and environmental cues and individual family members are induced or repressed under certain conditions. The combinatorial interaction between SAD/DUF581 proteins with SnRK1 in a specific physiological context greatly increases the regulatory flexibility of SnRK1 signaling.

SnRK1 mediated transcriptional reprogramming is likely to occur through physical interaction with transcription factors or other components of the transcriptional machinery inside the nucleus (Cho et al., 2012). However, only a few nuclear targets of SnRK1 have yet been identified that may act downstream of SnRK1 (Baena-González et al., 2007; Kleinow et al., 2009; Tsai and Gazzarrini, 2012; Chen et al., 2013b). In accordance with previous findings (Bitrian et al., 2011; Cho et al., 2012) a AKIN10-mCherry fusion protein displayed a nuclear-cytoplasmic distribution similar to the localization of GFP tagged SAD/DUF581 proteins. Upon co-expression, mCherry as well as GFP fluorescence was mainly detectable in nuclei of plants cells suggesting active re-localization of both proteins. In some cases the fluorescence signal was confined to discrete regions inside the nucleus. The *in planta* BiFC analyses further showed that interaction between AKIN10/11 and SAD/DUF581 proteins occurs in sub-nuclear foci. The nature of these sub-nuclear foci is currently unknown but it is conceivable that the spatial organization of SnRK1 signaling components and transcription factors into confined structures constitutes a regulatory strategy for modulating the activity of specific cellular pathways. In animal cells, many subnuclear foci containing transcription factors have been described, such as nuclear stress bodies, histone locus bodies, and polycomb bodies (Mao et al., 2011). Thus, it is likely that these structures within the nucleus implement important layers of regulation rather than being non-functional protein aggregates.

The observation that SAD/DUF581 proteins interact with SnRK1 raises the question as to whether they constitute SnRK1 phosphorylation targets or if they can act as scaffold proteins mediating SnRK1 binding to specific substrates. Although the former possibility can currently not be excluded we cautiously favor the latter. In this scenario, SAD/DUF581 proteins could interact with a third partner via their variable region and thus would bring potential substrates in close proximity to SnRK1 that otherwise cannot be recognized or do only weakly interact with the kinase. In our study, we identified STKR1 as an interaction partner of DUF581-18 in yeast. The founding member of this family of transcription factors has originally been described to regulate sucrose-inducible expression of patatin in potato tubers (Zourelidou et al., 2002). A recent study demonstrates that overexpression of an *Arabidopsis* storekeeper protein in tobacco prolongs the vegetative growth phase and delays flowering, suggesting a role for these proteins in the regulation of growth and developmental phasing (Bömer et al., 2011). The yeast two-hybrid data suggest that SnRK1 can interact with STKR1 independently of DUF581-18 in yeast and thus a mediator function of DUF581-18 would not be required for STKR1 to act as a substrate for SnRK1. Future experiments will have to investigate whether all three proteins are assembled into the same complex and whether DUF581-18 has the potential to modify the interaction between SnRK1 and STKR1 *in vivo*.

There are currently no genetic data demonstrating a role of SAD/DUF581 proteins in SnRK1 signaling and molecular studies on this protein family are scarce. DUF581-19 was previously described as MARD1 (MEDIATOR OF ABA-REGULATED DORMANCY 1) because a T-DNA insertion within the promoter region of the gene rendered seeds of the respective *Arabidopsis* plants insensitive toward ABA (He and Gan, 2004). In addition, *mard1/duf581-19* seeds displayed reduced dormancy and light-independent germination. This is reminiscent of seeds from SnRK1 antisense pea plants which also have been described to display phenotypic alterations related to ABA-insensitivity (Radchuk et al., 2006). Thus, the phenotype of *mard1/duf581-19* seeds could reflect perturbations in ABA mediated SnRK1 signaling. In turn, a DUF581 encoding gene has recently been described to be induced by salt stress in wheat (*Triticum aestivum*). Over-expression of the protein in transgenic *Arabidopsis* plants increased resistance toward abiotic stresses such as salinity and drought stress (Hou et al., 2013). Seeds of these plants were ABA-hypersensitive and root growth of seedlings could be inhibited by ABA treatment. These phenotypes could be explained by uncontrolled activation of SnRK1 signaling owing to the constitutive presence of the DUF581 containing protein from wheat.

In a recent study DUF581-18 has been shown to increase resistance toward aphids when over-expressed in *Arabidopsis*, while a knock-out of the corresponding gene promoted aphid infestation (Chen et al., 2013a). The underlying mechanism is currently unclear but it is interesting to note that constitutive overexpression of DUF581-18 resulted in smaller rosette leaves, delayed bolting time and smaller size of flowers and siliques. Thus, DUF581-18 might be involved in the regulation of growth, possibly through its interaction with STKR1 or related proteins.

Taken together, the results presented here strongly support a model in which SAD/DUF581 proteins act as components of SnRK1 signaling in plants by bridging the interaction of SnRK1 with specific target proteins. Conditional expression of SAD/DUF581 proteins enables SnRK1 signaling to respond to many different hormonal and environmental cues with specific cellular outputs. Binding to SAD/DUF581 proteins might also be involved in the regulation of SnRK1 by metabolites such as trehalose-6-phosphate.

The present study provides a framework for a functional analysis of SAD/DUF581 proteins in the context of SnRK1 signaling in plants. Future experiments will have to identify the interaction partners for each SAD/DUF581 protein and will seek to establish their relation to SnRK1 on the biochemical level. Detailed genetic studies using SAD/DUF581 over-expression and knock-out lines are necessary to investigate their role in SnRK1 signaling on the molecular level.

## Conflict of interest statement

The authors declare that the research was conducted in the absence of any commercial or financial relationships that could be construed as a potential conflict of interest.

## Supplementary material

Supplementary Figure S1 **Topology of DUF581 proteins from *Arabidopsis thaliana***.

Supplementary Figure S2 **Multiple sequence alignment of *Arabidopsis* DUF581 proteins**.

Supplementary Figure S3 **Interaction of DUF581-6-like from *Solanum tuberosum* with StubSNF1 in the yeast two-hybrid system**.

Supplementary Figure S4 **Controls for BiFC experiments**.

Supplementary Table S1 ***Arabidopsis* proteins annotated in TAIR to contain a DUF581**.

Supplementary Table S2 **Nucleotide sequences of primers used in this study**.
