# Corrigendum: The complex becomes more complex: protein-protein interactions of SnRK1 with DUF581 family proteins provide a framework for cell- and stimulus type-specific SnRK1 signaling in plants

**DOI:** 10.3389/fpls.2014.00693

**Published:** 2014-12-05

**Authors:** Frederik Börnke

**Affiliations:** ^1^Division of Biochemistry, Department of Biology, Friedrich-Alexander-Universität Erlangen-NürnbergErlangen, Germany; ^2^Plant Metabolism Group, Leibniz-Institute of Vegetable and Ornamental Crops (IGZ)Großbeeren, Germany; ^3^Institute of Biochemistry and Biology, University of PotsdamPotsdam, Germany

**Keywords:** Arabidopsis, SnRK1, DUF581, protein-protein interaction, stress signaling, ABA

The article “The complex becomes more complex: protein-protein interactions of SnRK1 with DUF581 family proteins provide a framework for cell- and stimulus type-specific SnRK1 signaling in plants” that is part of the research topic Trehalose and SnRK signaling pathways: integrators of growth, development and plant stress responses, published 21 February 2014, with myself as a corresponding author displays my affiliation incorrectly.

The correct author information is as follows:

Frederik Börnke^1,2,3^

^1^Division of Biochemistry, Department of Biology, Friedrich-Alexander-Universität Erlangen-Nürnberg, Erlangen, Germany

^2^Plant Metabolism Group, Leibniz-Institute of Vegetable and Ornamental Crops (IGZ), Großbeeren, Germany

^3^Institute of Biochemistry and Biology, University of Potsdam, Potsdam, Germany

## Conflict of interest statement

The author declares that the research was conducted in the absence of any commercial or financial relationships that could be construed as a potential conflict of interest.

