# Supplementary material for: The complex becomes more complex: protein-protein interactions of SnRK1 with DUF581 family proteins provide a framework for cell- and stimulus type-specific SnRK1 signaling in plants
Source: Front Plant Sci. 2014 Feb 21;5:54. doi: 10.3389/fpls.2014.00054 (PMC3930858; doi:10.3389/fpls.2014.00054)
Supplement: Supplementary Figure S1 — Topology of DUF581 proteins from Arabidopsis thaliana. [file DataSheet1.ZIP › Supplementary_Figure_S1.PDF]

Supplementary Figure S1

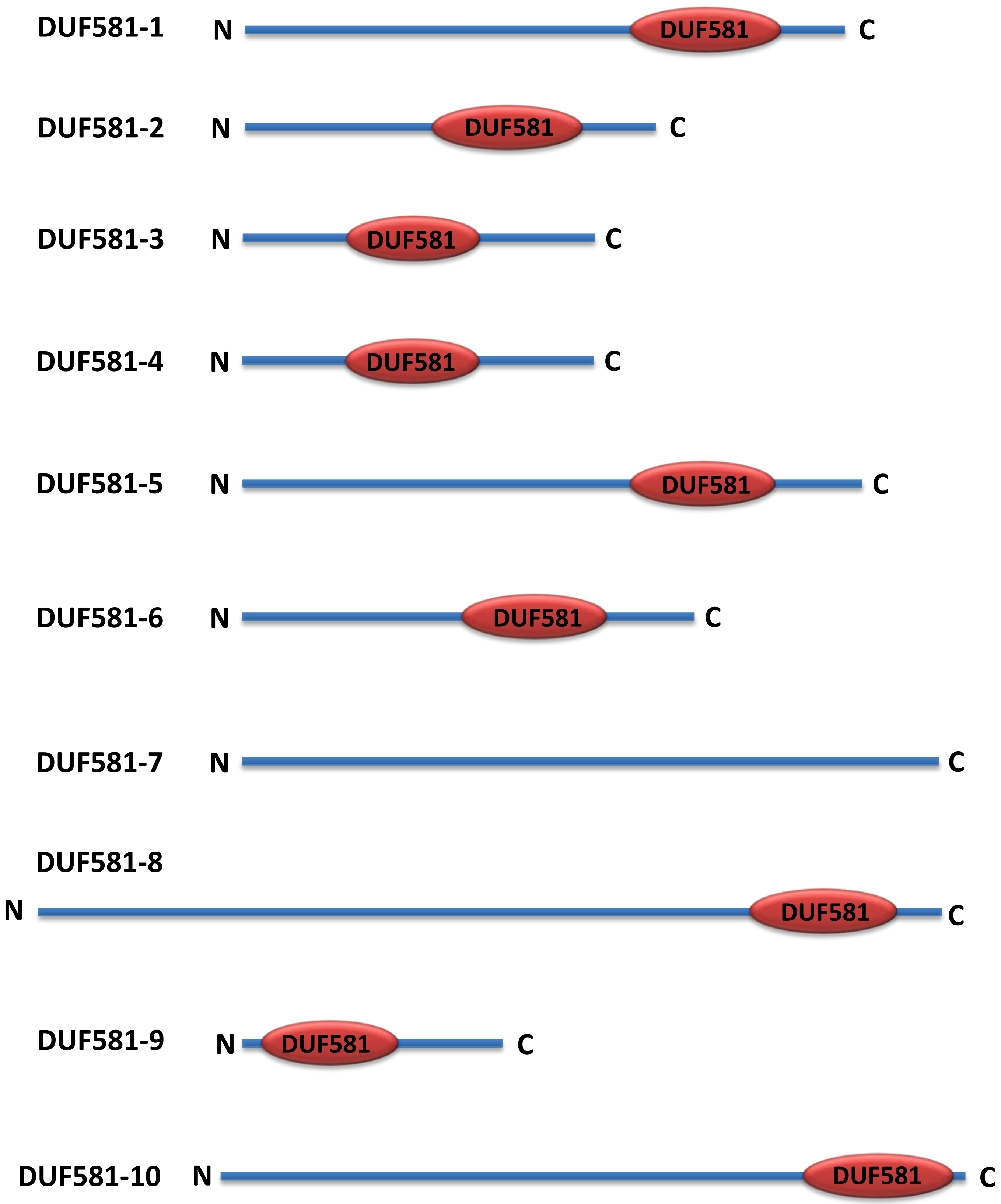

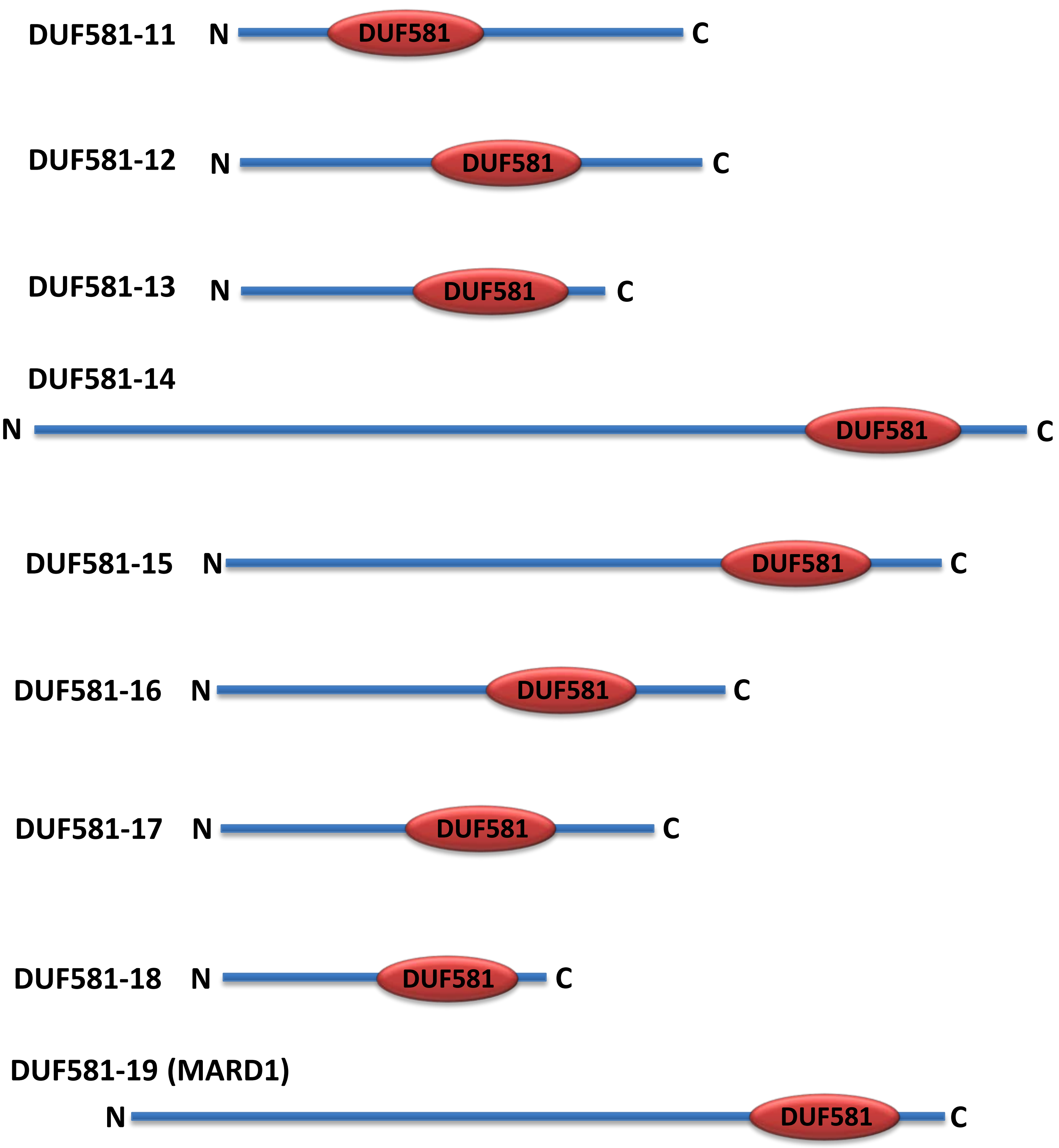

Supplementary Figure S1: Topology of DUF581 proteins from *Arabidopsis thaliana*. The position of the DUF581 on the polypeptide chain is indicated. 1 cm = 20 amino amino acids.
