# Supplementary material for: The complex becomes more complex: protein-protein interactions of SnRK1 with DUF581 family proteins provide a framework for cell- and stimulus type-specific SnRK1 signaling in plants
Source: Front Plant Sci. 2014 Feb 21;5:54. doi: 10.3389/fpls.2014.00054 (PMC3930858; doi:10.3389/fpls.2014.00054)
Supplement: Supplementary Figure S1 — Topology of DUF581 proteins from Arabidopsis thaliana. [file DataSheet1.ZIP › Supplementary_Figure_S2.pdf]

Supplementary Figure S2

```
DUF581-1      1 ---------------------------------------------MVVPGK-NSTISPDY
DUF581-2      1 ------------------------------------------------------------
DUF581-3      1 ------------------------------------------------------------
DUF581-4      1 ------------------------------------------------------------
DUF581-5      1 ----------------------------MILSKRPHLMIRKLSEMLVPRSR-SAAIKPEE
DUF581-6      1 ------------------------------------------------------------
DUF581-7      1 --------------MGEASDSDS--------SDESLKHNSTPPLFSISGFFCVAKGGDSD
DUF581-8      1 --MLKTRAMFPHKDQAMSLSLDPQSDLVVGHTNNRPITNPLALSLLIGLNNKNKCISDSD
DUF581-9      1 ------------------------------------------------------------
DUF581-10     1 --MLKKRSRSKQALMAETNQSQ-----------NQKQSKTTPFPRLFTAFSSFKSFTEND
DUF581-11     1 ----------------------------------------------------------MA
DUF581-12     1 ------------------------------------------------------------
DUF581-13     1 ------------------------------------------------------------
DUF581-14     1 --------MSQHSNYQMTTASDYYS--------TKPVLSAIRSHKLISSVFEGKCPSDYE
DUF581-15     1 -----------------------------MLTKRTHPMIGKISELLVGVNRSTAAPFFDV
DUF581-16     1 ------------------------------------------------------------
DUF581-17     1 ---------------------------------------MVGLSIVLEMTNNNNNNNNNN
DUF581-18     1 ------------------------------------------------------------
DUF581-19     1 MLRNKPRAAVTTKKQTSLLMADQPPPPKPNTCHCSPSLFSSPKFRFFTSKMMMTPFDSDF


DUF581-1     15 FTASQTSPLDMMKFPSPG---SSKRYDN---GGGIGLGIVAALEKSSIG--INPVCHTGA
DUF581-2      1 MLLGKRQRPPIKRTTSLS------------------------------------------
DUF581-3      1 MTKISVGLQLVTRDS---------------------------------------------
DUF581-4      1 MTKISVGLQLVTRDS---------------------------------------------
DUF581-5     32 YTASPRSPLDLN-FPSPV---HSKRFGS---GG-VGLGIVAALEETSNG--IN-------
DUF581-6      1 MLLGKRQRPPINRTTSLS------------------------------------------
DUF581-7     39 PGRSPTSPLDFGVFGGLFSPRSSSSSPLHRNKWLSHKVGLSLLSSSFELG----------
DUF581-8     59 FVRSPKSPLEFRVLSTMADSFFLRSPRSSLTAHLNCCCGPAAKVGLSIVDSLGDDRCLLP
DUF581-9      1 --MASYYSGFLG------------------------------------------------
DUF581-10    48 AVASPTSILDTKPFSVLKNPFGSDNPKTQEPETR--LKLEPKRIGLAIVDSLIQDETPEP
DUF581-11     3 NIMIPSKRPRAPSFSEKHP-----------------------------------------
DUF581-12     1 MEVSMRKPYFIEEEDDG-------------------------------------------
DUF581-13     1 MLLGNRPRPQMQRTASIT------------------------------------------
DUF581-14    45 SAWSPTSPLDFRLFSTLGNPFAASSSRSIWRGKQ--RSWDSGKVGLSIVHSLVDDHHTDS
DUF581-15    32 LMTSPKSPLDFKILPQISQRNSSKRFYDDNLGGSVGLGIVAALENSNTRRITSVCRSEPN
DUF581-16     1 MELSSRKPYFIEEEEEEN------------------------------------------
DUF581-17    22 NNNNNKNPLSEGVLISPK------------------------------------------
DUF581-18     1 MVLGKRHGSLIKRTTSMK------------------------------------------
DUF581-19    61 SLVSPTSILEANPSIFSSKNPKPVSYFEPTIPNPQRFHSPDVFGLADLVKDGDSNRDHSR


DUF581-1     67 GSKGFD------------------------------LARYSKRFQFAAGID----LSDSE
DUF581-2     19 ------------------------------------------EIKFDLNQPSEQ-EPSDH
DUF581-3     16 ------------------------------------------------------------
DUF581-4     16 ------------------------------------------------------------
DUF581-5     75 ---RHD------------------------------PVRYSGRFRCP-EID----LSD-E
DUF581-6     19 ------------------------------------------EIKFDLNLPSES-EPSNQ
DUF581-7     89 ----------------------------------------DDPFRRDYIVLAPQVKVNNV
DUF581-8    119 DIVFGP------ALRIKCSEVMDKHPKLLFPVANKSKKIENERSGVVFEIGDNSSETEPV
DUF581-9     11 ------------------------------------------------------------
DUF581-10   106 GPRSGT---------------------ILFGSQLRIRVPDSPISSSDFGIKTRNSQPETK
DUF581-11    22 ------------------------------------------------------------
DUF581-12    18 ---------------------------------------FVSLSEMEAGVSSPSCYNYPQ
DUF581-13    19 ------------------------------------------RITIEVDGDQTAGQDSDV
DUF581-14   103 SATIVLPSPDSKNIIFGSLMRSGQKPHLLSQPFTKALMPKDVIPNAVFEIGHDVIDVLEL
DUF581-15    92 QPGRSD------------------------------PVQFMSHGGSTDGEDEEMFIMDEE
DUF581-16    19 --------------------------------------LASSLSEMEAGFSGNNNNSNNH
DUF581-17    40 ------------------------------------------------------------
DUF581-18    19 ------------------------------------------MITLDTPTIYDASQPSDH
DUF581-19   121 KPVNKMVLFG---------------------------SKLRVQIPSSADFGTKTGIRYPP
```

```
DUF581-1    93 EYTCVTTRDGL----TKVYYKEEEFEFGHNLLNGDQRWRKPIEIAEESPAKERRVLRD--
DUF581-2    36 QIQLVN-----------------VDEHRQVHQRLLDQRLLAMVSPRGTQRRHSSDYSE--
DUF581-3    16 -----------------------------------REKLNNIVIKSSLRLNRSNPNIS--
DUF581-4    16 -----------------------------------REKLNNIVIKSSLRLNRSNPNIS--
DUF581-5    96 EYTYVTSPNGP----TKVYYNDDGFELSE---NDYRRVHKPMVTVDEPPVIERQSVRG--
DUF581-6    36 QKPTVASPYGS--------NGQAVTAAVDQNRGFLDQRLLSMVTPRGNLRRHSGDFSD--
DUF581-7   109 NTATPKLSSDPCVIVEEPRRSSSSSPMDIISTYSRSLSGREMALSEDYTCIISHGPNPK-
DUF581-8   173 GLRNRSFSANDCLRKTRVLSRSKLGQEGDFPGSGSDNAFSSEDDMEDYTCIIAHGPNPKT
DUF581-9    11 --------------------------------------------------------CE--
DUF581-10  145 KPGSESGLG------------SPRIISGYFPAS-------DMELSEDYTCVTCHGPNPRT
DUF581-11   22 -----------------------------------KYVGSSDWLPAEKDKAQVQLTN---
DUF581-12   39 SYYYN---------------------HHHHQYSVSSPR---SGKFHDFRFDNSYYGYG--
DUF581-13   37 SMTVVD------------------------GGENYAQRFLSPVNHQRNERKYGGRSSP--
DUF581-14  163 RKSGSVDAAYCSGAENFSVNNNACQVTKQDPGSLNGGTESDMEISEDYTCVISHGPNPKT
DUF581-15  122 DYTLVTCHHGPSGSCNTRVYDKDGFECFSSKINDDRRERLFVVDVVTE-SPENSPEFQ--
DUF581-16   41 GNPQNGVVSSSRFSYGRLNSLRNSQSYYYNQYSVSSPRSVVSGRFHDFRFD-----IQ--
DUF581-17   40 ------------------------------VVNKANIIVTTAVTTDTTNLRRCYQDSG--
DUF581-18   37 --------------------------------------LTFHQHPHNPMVVMASNYDD--
DUF581-19  154 CQLSPCVQTKVLAVSEIDQTEDYTRVISHGPNPTITHIFDNSVFVEATPCSVPLPQPAME

DUF581-1   147 ---------------------------------------------CPDFLTSCCLCKKKL
DUF581-2    77 ---------------------------------------------D--FLRSCSLCKRLL
DUF581-3    39 ---------------------------------------------ELCFLKTCHLCNKQL
DUF581-4    39 ---------------------------------------------ELCFLKTCHLCNKQL
DUF581-5   147 ---------------------------------------------PTEFLSSCCLCKKKL
DUF581-6    86 ---------------------------------------------AGHFLRSCALCERLL
DUF581-7   168 --------------------------------------------TTYIFGDCILDCDPKD
DUF581-8   233 THIYGDRVLECHKN-ELKGDEDNKEK-------------FGSVFPSDNFLGICNFCNKKL
DUF581-9    13 ---------------------------------------------EPHFLESCSLCRKHL
DUF581-10  186 IHIFDNCIVESQPGVVFFRSSDPVNE------------SDSDYSPPDSFLSCCCNCKKSL
DUF581-11   44 ------------------------------------------------FLELCRFCKKNL
DUF581-12   73 ---------------------------------------------QPHFLDSCFLCKKRL
DUF581-13   71 ---------------------------------------------SS-FLVNCGFCKRGL
DUF581-14  223 THFYGDQVMESVEREELKNRCCKNEKESIFAVAPLDLTTPVDVLPPKDFLSFCYGCSKKL
DUF581-15  179 ---------------------------------------------GLGFLNSCYLCRKKL
DUF581-16   94 ---------------------------------------------QPHFLDSCFLCKKPL
DUF581-17   68 ------------------------------------------------FLEHCFLCRRKL
DUF581-18   57 ------------------------------------------------FLKTCSLCNRSL
DUF581-19  214 TKS------------------------------------------TESFLSRCFTCKKNL

DUF581-1   162 Q-GKDIYMYK-GDEGFCSKECRSLKIMEDSLKE---------QHKLTS-------VEVLT
DUF581-2    90 VHGRDIYMYR-GDRAFCSLECRQQQITVDERKE---------KKKGSVRSTIVVATGTTT
DUF581-3    54 HQDKDVYMYR-GDLGFCSRECRESQMLIDDRKELEASTKMMLASYRRCNNGAGKSESRNL
DUF581-4    54 HQDKDVYMYR-GDLGFCSRECRESQMLIDDRKELEASTKMMLASYRRCNNGAGKSESRNL
DUF581-5   162 Q-GKDIYMYK-GEMGFCSAECRSVQIMNDERQE---------QCKTQVSRNADVLSSPYA
DUF581-6   101 VPGRDIYMYR-GDKAFCSSECRQEQMAQDERKE---------KGKSAAPAKEPAVTAPAR
DUF581-7   184 LGKEDIEIHEEGDDSFSTEKPQNHREVSAEKESENAGAEESCYEEEDLFPMAMPLNP---
DUF581-8   279 GGGDDIYMYR--EKSFCSEECRSEEMMIDEED---LEEPCIDMHESLKKLF---------
DUF581-9    28 GLNSDIFMYR-GDKAFCSNECREEQIESDEAKERK------WKKSSRSLRKNSSETKESA
DUF581-10  234 GPRDDIFMYR-GDRAFCSSECRSIEMMMSEENDTK-------------------------
DUF581-11   56 RHDEDVFMYG-YLGAFCSKQCRAKQMACDVFRDFSRQKANCRWMCATIKPFASFFKGNWL
DUF581-12   88 GDNRDIFMYR-GDTPFCSEECREEQIERDEAKEKKQSLSTSVKAMRRNEKRSSSSSPTRS
DUF581-13   85 APGRDIYMYK-GDAAFCSIECREQQMEHDEGKT---------RNRVVLSPSK--------
DUF581-14  283 GMGEDIYMYS-GYKAFCSSECRSKEIDLDEEMEDGDEEEAIKSVSSSDKESKKKSNGVFF
DUF581-15  194 H-GQDIFIYR-GEKAFCSTECRSSHIANDERKE---------RCRSKFS------TSPYT
DUF581-16  109 GDNRDIYMYR-GDTPFCSEECRQEQIERDEAKEKKQNLSHSVKSAMR---RKEQSSPTRS
DUF581-17   80 LPAKDIYMYK-GDRAFCSVECRSKQMIMDEEESLRREYCSLMDVKKKKFDSPATAPSRYR
DUF581-18   69 CHHRDIYMYR-GNNAFCSLECREKQIKLDEKKA---------KTGFVTSKKPIRI-----
DUF581-19  232 DQKQDIYIYR-GEKGFCSSECRYQEMLLDQMET---------------------------
```

```
DUF581-1   204 GEEIASPGIFLI-----------------------------
DUF581-2   140 G----ERVSAAV-----------------------------
DUF581-3   113 FDDLRRRRQLFIVP---------------------------
DUF581-4   113 FDDLRRRRQLFIVP---------------------------
DUF581-5   211 AGQRLSAGVFVF-----------------------------
DUF581-6   151 AKPGKGRAAAAV-----------------------------
DUF581-7       -----------------------------------------
DUF581-8       -----------------------------------------
DUF581-9    81 AGNTVRTGTLVVA----------------------------
DUF581-10      -----------------------------------------
DUF581-11  115 TTLTSLHRHIIICRSNFLFGYLGFSFFSLLCITNLMYKCVF
DUF581-12  147 RNYAFRTGTVAAA----------------------------
DUF581-13      -----------------------------------------
DUF581-14  342 TVG--------------------------------------
DUF581-15  237 AGQIFSTGVLVT-----------------------------
DUF581-16  165 RDYAFHNGTVAAA----------------------------
DUF581-17  139 RDPRNQAGGFAY-----------------------------
DUF581-18      -----------------------------------------
DUF581-19      -----------------------------------------
```

Supplementary Figure S2: Multiple sequence alignment of Arabidopsis DUF581 containing proteins. Sequences were aligned using ClustalW and conserved amino acid residues were highlighted using Boxshade.
