# Supplementary material for: The complex becomes more complex: protein-protein interactions of SnRK1 with DUF581 family proteins provide a framework for cell- and stimulus type-specific SnRK1 signaling in plants
Source: Front Plant Sci. 2014 Feb 21;5:54. doi: 10.3389/fpls.2014.00054 (PMC3930858; doi:10.3389/fpls.2014.00054)
Supplement: Supplementary Figure S1 — Topology of DUF581 proteins from Arabidopsis thaliana. [file DataSheet1.ZIP › Supplementary_Figure_S3.PDF]

Supplementary Figure S3.

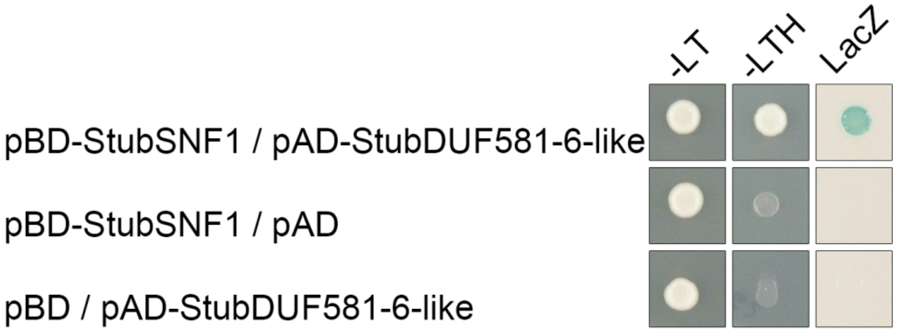

Supplementary Figure S3. Interaction of potato SnRK1 (StubSNF1) with a DUF581 containing protein (StubDUF581-6-like) from potato in yeast two-hybrid assays.

StubSNF1 fused to the GAL4 DNA binding domain was expressed in combination with StubDUF581-6-like fused to the GAL4 activation domain (AD) in yeast strain Y190. Cells were grown on selective media before a LacZ filter assay was performed. The empty AD vector served as negative control. –LT, yeast growth on medium without Leu and Trp. –LTH, yeast growth on medium lacking His, Leu, and Trp, indicating expression of the HIS3 reporter gene. LacZ, activity of the *lacZ* reporter gene.
