# Supplementary material for: The complex becomes more complex: protein-protein interactions of SnRK1 with DUF581 family proteins provide a framework for cell- and stimulus type-specific SnRK1 signaling in plants
Source: Front Plant Sci. 2014 Feb 21;5:54. doi: 10.3389/fpls.2014.00054 (PMC3930858; doi:10.3389/fpls.2014.00054)
Supplement: Supplementary Figure S1 — Topology of DUF581 proteins from Arabidopsis thaliana. [file DataSheet1.ZIP › Supplementary_Figure_S4.PDF]

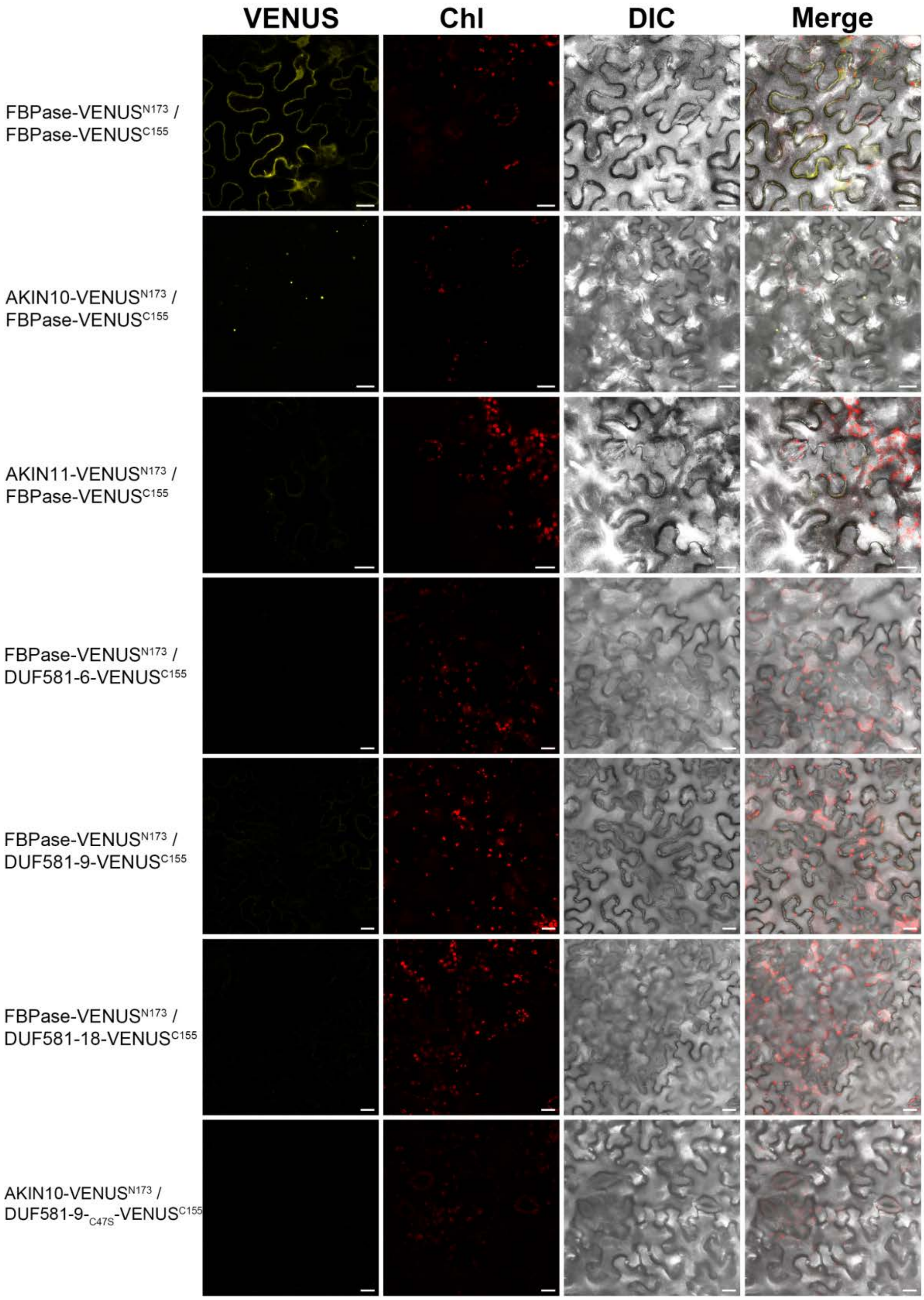

Supplementary Figure S4: Control experiments for BiFC assays. YFP confocal microscopy images show a merge YFP- and chlorophyll auto-fluorescence of tobacco leaf epidermal cells transiently expressing constructs encoding the fusion proteins indicated. Each image is the representative of at least three experiments.
