# Supplementary material for: The complex becomes more complex: protein-protein interactions of SnRK1 with DUF581 family proteins provide a framework for cell- and stimulus type-specific SnRK1 signaling in plants
Source: Front Plant Sci. 2014 Feb 21;5:54. doi: 10.3389/fpls.2014.00054 (PMC3930858; doi:10.3389/fpls.2014.00054)
Supplement: Supplementary Figure S1 — Topology of DUF581 proteins from Arabidopsis thaliana. [file DataSheet1.ZIP › Supplementary_Table_S1.pdf]

Supplementary Table S1. Arabidopsis proteins annotated in TAIR to contain a DUF581

Name	AGI Code	Length (aa)	Postion of DUF581	No. of associated ESTs
DUF581-1	At1g19200	215	138-192	3
DUF581-2	At1g22160	147	67-121	61
DUF581-3	At1g53885	126	36-85	10
DUF581-4	At1g53903	126	36-85	4
DUF581-5	At1g74940	222	140-192	81
DUF581-6	At1g78020	162	78-132	76
DUF581-7	At1g79970	250	n.d.	56
DUF581-8	At2g25690	324	254-307	6
DUF581-9	At2g44670	93	10-59	103
DUF581-10	At3g22550	267	210-264	26
DUF581-11	At3g63230	155	31-84	0
DUF581-12	At4g17670	159	67-119	23
DUF581-13	At4g39795	126	60-115	3
DUF581-14	At5g11460	344	265-314	6
DUF581-15	At5g20700	248	170-224	102
DUF581-16	At5g47060	177	89-140	32
DUF581-17	At5g49120	150	59-110	4
DUF581-18	At5g65040	113	50-99	12
DUF581-19	At3g63210	263	211-262	54
