# Supplementary material for: The complex becomes more complex: protein-protein interactions of SnRK1 with DUF581 family proteins provide a framework for cell- and stimulus type-specific SnRK1 signaling in plants
Source: Front Plant Sci. 2014 Feb 21;5:54. doi: 10.3389/fpls.2014.00054 (PMC3930858; doi:10.3389/fpls.2014.00054)
Supplement: Supplementary Figure S1 — Topology of DUF581 proteins from Arabidopsis thaliana. [file DataSheet1.ZIP › Supplementary_Table_S2.pdf]

**Supplemental Table 2: Nucleotide sequences of gene-specific primers.**		
Nucleotide sequence of gene-specific primers for the construction of pENTR-clones		
DUF581-1	Fw	5'- CACCAACAAAATGGTGGTTCCAGGTAAAAACTCC-3'
	Rv	5'- TATAAGGAATATTCCGGGGGAGGCAAT-3'
DUF581-2	Fw	5'-CACCAACAAAATGTTGCTAGGGAAGAGACAGAGA-3'
	Rv	5'-CACGGCGGCGGAAACTCTCTCTCCT-3'
DUF581-3	Fw	5'- CACCAACAAAATGACTAAAATCTCTGTTGGATTG-3'
	Rv	5'-AGGAACTATAAATAATTGACGTCG-3'
DUF581-4	Fw	5'-CACCAACAAAATGACTAAAATCTCTGTTGG-3'
	Rv	5'-AGGAACTATAAATAATTGACGTC-3'
DUF581-5	Fw	5'-CACCAACAAAATGATACTAAGCAAGAGACCT-3'
	Rv	5'-AAATACGAATACTCCGGCAGATAATC-3'
DUF581-6	Fw	5'-CACCAACAAAATGTTGCTTGGAAAGAGACAAC-3'
	Rv	5'-CACGGCGGCTGCAGCTCTCCCTTTG-3'
DUF581-7	Fw	5'-CACCAACAAAATGGGTGAGGCAAGTGATTCAGA-3'
	Rv	5'-CGGGTTAAGAGGCATTGCCA-3'
DUF581-8	Fw	5'-CACCAACAAAATGCTGAAGACAAGAGCAATG-3'
	Rv	5'-GAAGAGCTTCTTTAAAGATTCA-3'
DUF581-9	Fw	5'-CACCAACAAAATGGCTTCGTATTACTCT-3'
	Rv	5'-AGCCACGACGAGAGTTCCTG-3'
DUF581-10	Fw	5'-CACCAACAAAATGCTAAAGAAGAGATCAAG-3'
	Rv	5'-TTTAGTATCATTTTCCTCTG-3'
DUF581-11	Fw	5'-CACCAACAAAATGGCGAATATAATGATTCC-3'
	Rv	5'-GAAAACACACTTGTACATCAAATTC-3'
DUF581-12	Fw	5'-CACCAACAAAATGGAGGTTTCGATGAGAAAG-3'
	Rv	5'-GGCAGCAGCAACAGTTCCAGTG-3'
DUF581-13	Fw	5'-CACCAACAAAATGTTGTTAGGGAATCGACC-3'
	Rv	3'-TTTTGAAGGTGATAGTACGAC-3'
DUF581-14	Fw	5'-CACCAACAAAATGTCTCAGCATTCCAACTATC-3'
	Rv	5'-GCCCACGGTGAAGAACACACC-3'
DUF581-15	Fw	5'-CACCAACAAAATGCTTACTAAAAGAACCC-3'
	Rv	5'-AGTCACTAGAACTCCGGTGG-3'
DUF581-16	Fw	5'-CACCAACAAAATGGAGCTTTCATCGAGAAAG-3'
	Rv	5'-AGCAGCAGCAACAGTTCCATTG-3'
DUF581-17	Fw	5'-CACCAACAAAATGGTGGGACTAAGTATTGT-3'
	Rv	5'-GTAAGCGAAACCGCCTGCTTG-3'
DUF581-18	Fw	5'-CACCAACAAAATGGTGTTAGGAAAGCGTCA-3'
	Rv	5'-AATACGAATTGGTTTCTTCGATG-3'
DUF581-19	Fw	5'-CACCAACAAAATGCTTAGAAACAAACC-3'
	Rv	5'-GGTCTCCATTTGATCAAGAAGC-3'
SnRK1.1/AKIN10	Fw	5'-CACCAACAAAATGTTCAAACGAGTAGATGAGTTTA-3'
	Rv	5'-GAGGACTCGGAGCTGAGCAAGAAAAGC-3
SnRK1.2/AKIN11	Fw	5'-CACCAACAAAATGGATCATTCATCAAATAGATTTG-3'
	Rv	5'-GATCACACGAAGCTCTGTAAGAAACC-3'
Nucleotide sequence of gene-specific primers for site-directed mutagenesis		
DUF581-9C47S	Fw	5'-GACAAGGCTTTTTGTAGCAACGAGTCTAGAGAAGAACAGATTGAATCT-3'
	Rv	5'-AGATTCAATCTGTTCTTCTCTAGACTCGTTGCTACAAAAAGCCTTGTC-3'
Nucleotide sequence of gene-specific primers for the construction of deletion clones		
DUF581-1Domain	Fw	5'-CACCAACAAATTTCTAACTTCTTGTTGC-3'
	Rv	5'-TCACTCTTTTAAACTATCCTCC-3'
